# Invasion by *Cedrela odorata* threatens long distance migration of Galapagos tortoises

**DOI:** 10.1002/ece3.10994

**Published:** 2024-02-13

**Authors:** Stephen Blake, Freddy Cabrera, Gonzalo Rivas‐Torres, Sharon L. Deem, Ainoa Nieto‐Claudin, Rakan A. Zahawi, Guillaume Bastille‐Rousseau

**Affiliations:** ^1^ Department of Biology Saint Louis University St. Louis Missouri USA; ^2^ Max Planck Institute of Animal Behavior Radolfzell Germany; ^3^ WildCare Institute Saint Louis Zoo Saint Louis Missouri USA; ^4^ Charles Darwin Foundation Puerto Ayora Galapagos Ecuador; ^5^ Ecuador Colegio de Ciencias Biológicas y Ambientales and Galapagos Academic Institute for the Arts and Sciences Universidad San Francisco de Quito Quito Ecuador; ^6^ Department of Wildlife Ecology and Conservation University of Florida Gainesville Florida USA; ^7^ Geography University of North Carolina at Chapel Hill Chapel Hill North Carolina USA; ^8^ Institute for Conservation Medicine Saint Louis Zoo Saint Louis Missouri USA; ^9^ School of Life Sciences University of Hawai'i at Mānoa Honolulu Hawaii USA; ^10^ Cooperative Wildlife Research Laboratory Southern Illinois University Carbondale Illinois USA

**Keywords:** alien species, conservation, ecosystem engineer, eradication, GPS telemetry, megaherbivores, silviculture, vegetation mapping

## Abstract

Invasive alien species are among the most pervasive threats to biodiversity. Invasive species can cause catastrophic reductions in populations of native and endemic species and the collapse of ecosystem function. A second major global conservation concern is the extirpation of large‐bodied mobile animals, including long‐distance migrants, which often have keystone ecological roles over extensive spatial extents. Here, we report on a potentially catastrophic synergy between these phenomena that threatens the endemic biota of the Galapagos Archipelago. We used GPS telemetry to track 140 migratory journeys by 25 Western Santa Cruz Island Galapagos tortoises. We plotted the spatial interaction between tortoise migrations and recently established non‐native forest dominated by the invasive tree *Cedrela odorata* (*Cedrela* forest). We qualified (a) the proportion of migratory journeys that traversed *Cedrela* forest, and (b) the probability that this observed pattern occurred by chance. Tortoise migrations were overwhelmingly restricted to small corridors between *Cedrela* forest blocks, indicating clear avoidance of those blocks. Just eight of 140 migrations traversed extensive *Cedrela* stands. Tortoises avoid *Cedrela* forest during their migrations. Further expansion of *Cedrela* forest threatens long‐distance migration and population viability of critically endangered Galapagos tortoises. Applied research to determine effective management solutions to mitigate *Cedrela* invasion is a high priority.

## Graphical abstract

Maintaining long‐distance elevational migration in the face of environmental change is important to ensure both the reproductive success and keystone ecological role of giant tortoises on Santa Cruz Island, Galapagos. Here, we show that rapid range expansion by *Cedrela odorata*, an invasive tree, threatens the last migration corridors of western Santa Crus Galapagos tortoises. Finding effective management solutions to *Cedrela* expansion is critical for conservation for critically endangered Galapagos tortoises and their ecosystem.

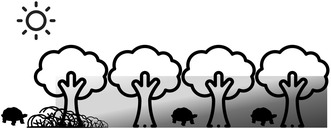

## 1 INTRODUCTION

Invasion by alien species threatens biodiversity conservation from local to global scales (Weidlich et al., 2020). Invasive species have profound evolutionary and ecological impacts including reducing native species abundance and diversity, modifying trophic interactions, changing the genetic structure of native populations, and altering the ecological function of communities (Pyšek et al., 2020). Alien invasive species are problematic throughout the world, but the biotas of oceanic islands are particularly vulnerable because of high levels of endemism and the poor competitive ability of many endemic species (Caujape‐Castells et al., 2010). The exponential growth of human transportation networks has facilitated the spread of exotic species globally and today, even on the most remote island archipelagos, invasive species outnumber endemic and native species (Heleno et al., 2013).

A second major global threat to biodiversity conservation is the extirpation of animals that move over large spatial extents, including long‐distance migrants such as large vertebrates. Megavertebrates are often the first to disappear from ecosystems due to their low densities and large home ranges, which often bring them into conflict with humans and human infrastructure that can impede access to high quality habitats (Blake et al., 2009). The most threatened vertebrates are large‐bodied, long‐distance migrants that tend to traverse biome boundaries, international borders, and/or human footprint gradients (Shuter et al., 2012). The loss of large migratory vertebrates can have profound consequences for biodiversity because these species typically play important roles in ecosystem function. For example, herbivory and trampling by large‐bodied vertebrates increases habitat heterogeneity and community composition (Blake et al., 2009), while migrants move seeds, spores, disease, and nutrients over long distances (Subalusky et al., 2017) that can shape habitat structure over greater spatial extents than do resident species. Accordingly, the decline or extirpation of these species may lead to reductions in community diversity and ecosystem function.

Here, we report on a potentially catastrophic synergy between these phenomena and that threatens the endemic biota of the Galapagos Archipelago. Specifically, we focus on how the distribution of an aggressive, alien, invasive tree species, *Cedrela odorata* (Meliaceae) (hereafter *Cedrela*), is jeopardizing one of the last intact terrestrial long‐distance migrations on earth—that of critically endangered Western Santa Cruz Galapagos tortoises (WCS tortoises, *Chelonoidis porteri*).

Since its introduction into highland farms on Santa Cruz during the 1940s, *Cedrela* has expanded beyond the agricultural areas and into the Galapagos National Park where it has had a devastating impact on native plant diversity. Rivas‐Torres, Benítez, et al. (2018) reported that forest invaded by *Cedrela* (“*Cedrela* forest”) had 42% and 17% lower endemic plant species richness and diversity, respectively, than non‐invaded endemic forest, while non‐native species richness and diversity were considerably higher in *Cedrela* forest (36% and 37%, respectively). *Cedrela* timber is now a valuable commodity for the local building and furniture industries on Galapagos, with an annual local market value estimated at $2 million in 2012 (Rivas‐Torres & Adams, 2018). Although, the rate of expansion of *Cedrela* on Santa Cruz has not been well quantified, dense and extensive patches of *Cedrela* forest, with few gaps of native vegetation between patches, now extend into the Galapagos National Park and dominate the park/agricultural zone boundary (Rivas‐Torres, Luke Flory, & Loiselle, 2018) (Figure 2).

**FIGURE 1:**
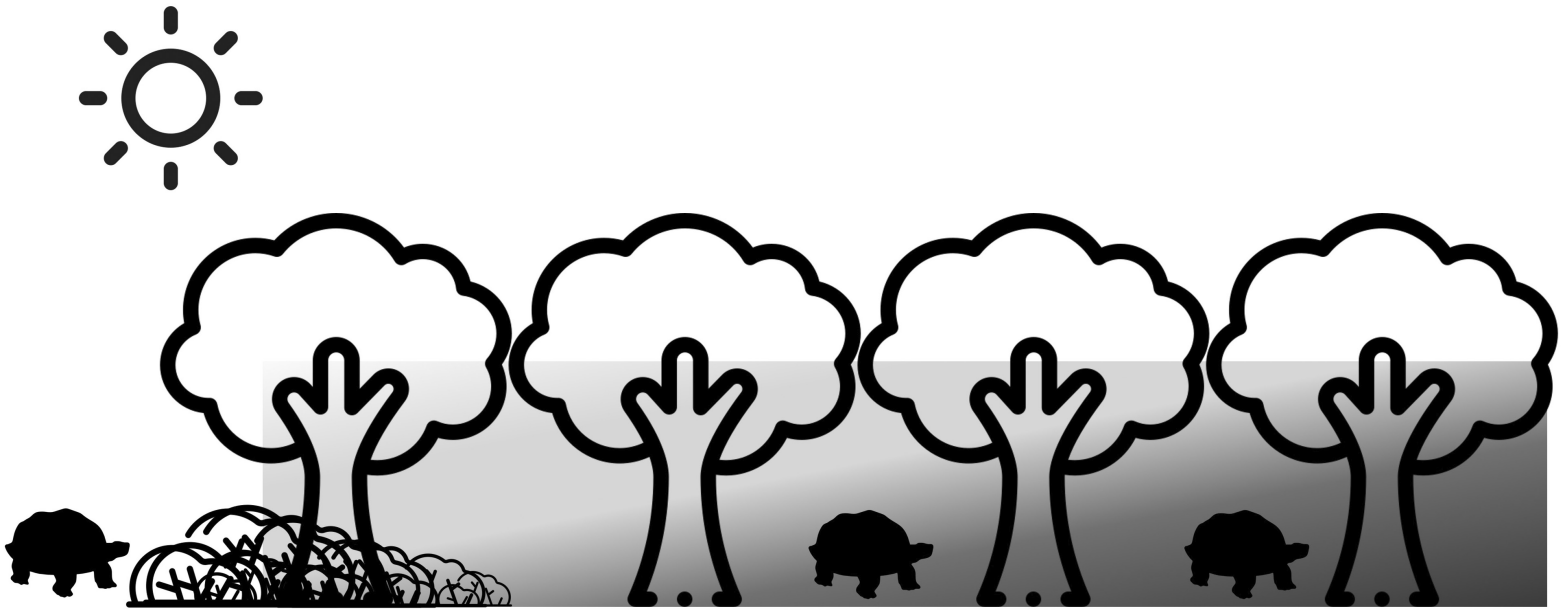
Conceptual model illustrating hypotheses on why Galapagos tortoises may avoid *Cedrela* forest on Santa Cruz Island. Edge effects at the *Cedrela* forest boundary promotes invasion by exotic blackberry, which often creates impenetrable thickets that block migratory Galapagos tortoises. With distance from the edge into the interior of large *Cedrela* forest patches shade increases, and tortoises have greater difficulty maintaining body temperature. Furthermore, low light levels coupled with the allelopathic nature of *Cedrela*, eliminates ground vegetation from the forest floor, limiting forage availability for tortoises.

Recent qualitative observations using raw Google Earth imagery suggest that consistent constrictions of tortoise migratory routes around a number of pinch points may be due to the distribution of invasive species, particularly *Cedrela* (Blake et al., 2021). We speculate that tortoises may be avoiding *Cedrela* for three reasons (Figure 1). First, the edges of *Cedrela* forest are often invaded by dense thickets of alien, invasive blackberry (*Rubus niveus*) that can be impenetrable to Galapagos tortoises. Second, tortoises may be unable to maintain body temperature in large, mature *Cedrela* stands, because the canopy of *Cedrela* forest is extremely dense which prevents solar radiation penetrating to ground level. Third, the lack of light on the forest floor and the allelopathic nature of *Cedrela* (Rivas‐Torres & Rivas, 2018) reduces ground vegetation, limiting foraging opportunities for tortoises.

**FIGURE 2:**
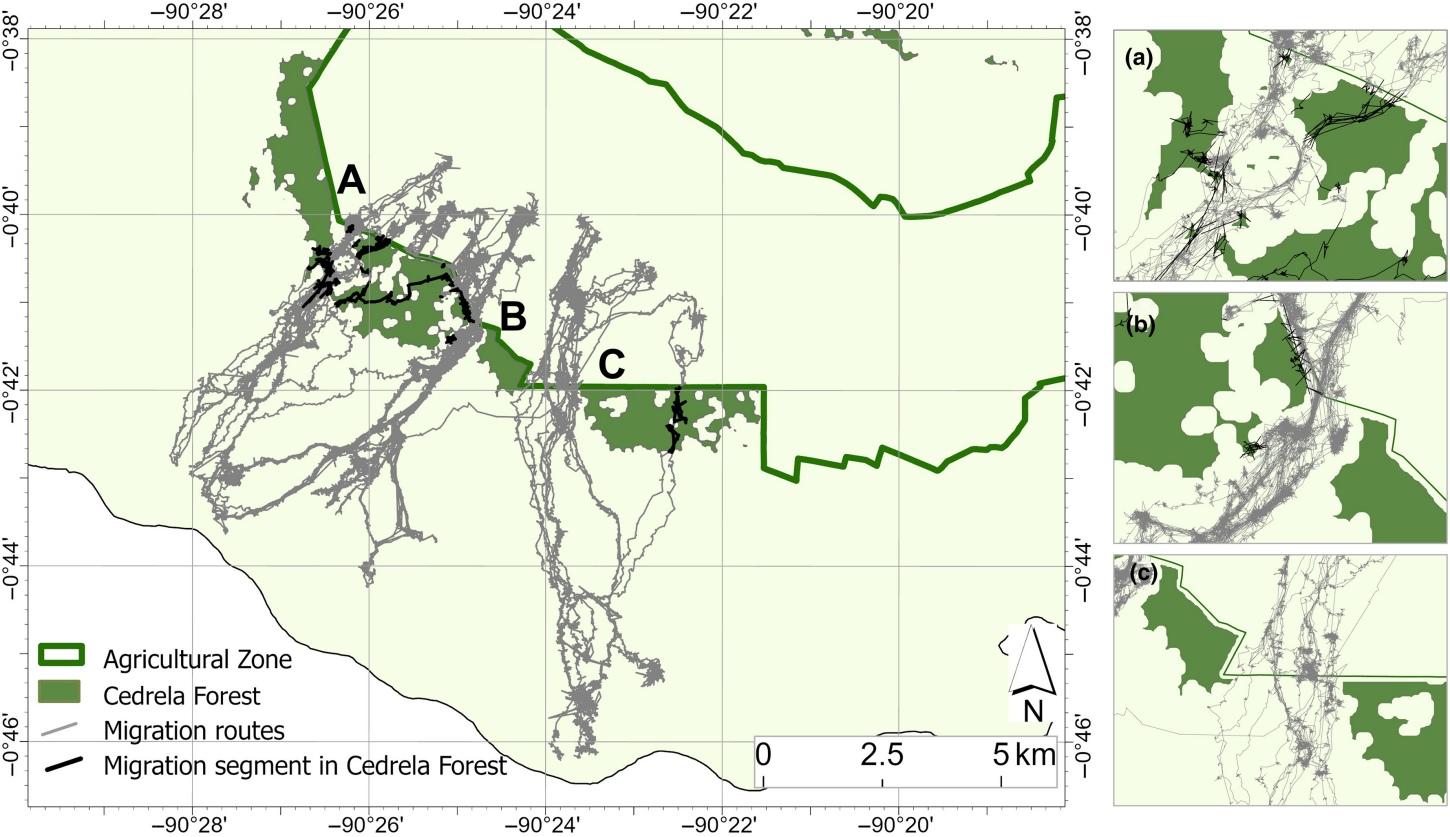
Map of the southwestern flank of Santa Cruz Island, Galapagos, showing *Cedrela* forest distribution (dark green) and 140 Western Santa Cruz tortoise migration routes (gray tracks). Black tracks are migration route segments that pass through *Cedrela* forest. The three gaps in *Cedrela* forest cover are labeled A, B, and C. Inserts to the right of the main figure illustrate the detail of tortoise tracks in relation to the three gaps in *Cedrela* forest cover.

Much of this *Cedrela* forest occurs in an area known as the Tortoise Reserve, so named because of the high abundance of WSC tortoises. Western Santa Cruz tortoises are partial long‐distance migrants, in which a portion of the population is migratory and the remainder sedentary or nomadic (Bastille‐Rousseau et al., 2016). Migrants follow seasonal patterns in the distribution of vegetation productivity along the elevation gradient of the island's southern flank (Bastille‐Rousseau et al., 2017) (Figure 2). During the cool dry season from June to December, Galapagos lowlands become increasingly arid, vegetation productivity declines in response, and forage availability for tortoises is low. However, persistent cloud cover in the Galapagos highlands maintains precipitation and high soil moisture allowing year‐round vegetation productivity. Migratory tortoises “follow the green” (Mueller et al., 2008) into the highlands when lowland vegetation declines (Blake et al., 2013). During the hot wet season, from January to May, high lowland rainfall promotes a vigorous green‐up of high quality forage and migratory tortoises return to the lowlands (Blake et al., 2013). Thus, migrants have year‐round access to high quality forage in both humid highlands during the cool dry season, and arid lowlands during the hot wet season. Adult females nest only in lowland areas on Santa Cruz Island (Blake et al., 2024). A recent bioenergetic model predicted that migratory tortoises should maintain a positive annual energy balance, while adult tortoises resident in the lowlands are expected to incur an energy deficit (Yackulic et al., 2017). Empirical evidence supports the theoretical model showing that migration improves tortoise body condition, and potentially fecundity, compared to residency (Blake et al., 2015, 2024). Given the well‐known negative consequences of anthropogenic disruption of long‐distance migrations on population persistence around the world (Wilcove & Wikelski, 2008), and for migratory tortoises on Galapagos (Blake et al., 2021), we became interested in the potential impact of environmental change on WSC tortoise migration and the broader implications for conservation.

Here, we report on the impact of *Cedrela* invasion of the Galapagos National Park on the migratory routes of WSC Galapagos tortoises by investigating the spatial relationships between tortoise migrations and *Cedrela* forest. We were most interested in determining to what extent migratory tortoises avoided entering or traversing *Cedrela* forest, and whether likely future expansion of *Cedrela* could jeopardize the migration.

## 2 METHODS

### 2.1 GPS telemetry of giant tortoises

Since 2009, we have been monitoring the movements of adult WCS tortoise using GPS telemetry (for details of GPS tag deployment and other methodological details, see Blake et al. (2021)). Twenty‐five adults were tracked for durations between 101 and 3959 days providing a total of 564,227 GPS fixes (Table S1) (all data are stored on movebank.org). All tagged adult tortoises were either migratory or nomadic (Bastille‐Rousseau et al., 2017). We quantified how many individual tortoises were impacted by *Cedrela*. To do so, we first extracted a shapefile of estimated *Cedrela* distribution from a vegetation map of Galapagos produced by Rivas‐Torres, Luke Flory, and Loiselle (2018). Given uncertainty in the GPS locations (estimated at ca. 15 m) and of the resulting map, we removed a 50 m buffer on the inside of *Cedrela* forest patches to estimate the proportion of individuals that traverse, and not just marginally overlap, *Cedrela* forest. We then used ArcGIS Pro to convert a point shapefile of all tortoise locations into a line shapefile (points to lines tool), and then used the intersect tool to determine when tortoise line trajectories traversed the boundary of *Cedrela* forest. We then tallied how many tortoise migratory journeys overlapped with *Cedrela* and how many journeys ended at the periphery of *Cedrela* forest.

We evaluated the probability of observing the current overlap of tortoise migratory journeys with *Cedrela* forest by chance. We performed a randomization approach similar to a path selection function (Zeller et al., 2016) where we compared the observed tortoise migratory journeys to randomized samples of those paths (i.e., the whole migratory trajectory was randomized). To do so, we randomly rotated and shifted the journeys and extracted the proportion of locations in each journey overlapped with *Cedrela* forest. Paths were randomly rotated by +/− 15 degrees and randomly shifted by up to 1 km in any direction (north, east, south, and west). We limited the angle of rotation and shift to preserve the migratory aspect of movement.

## 3 RESULTS

**FIGURE 3:**
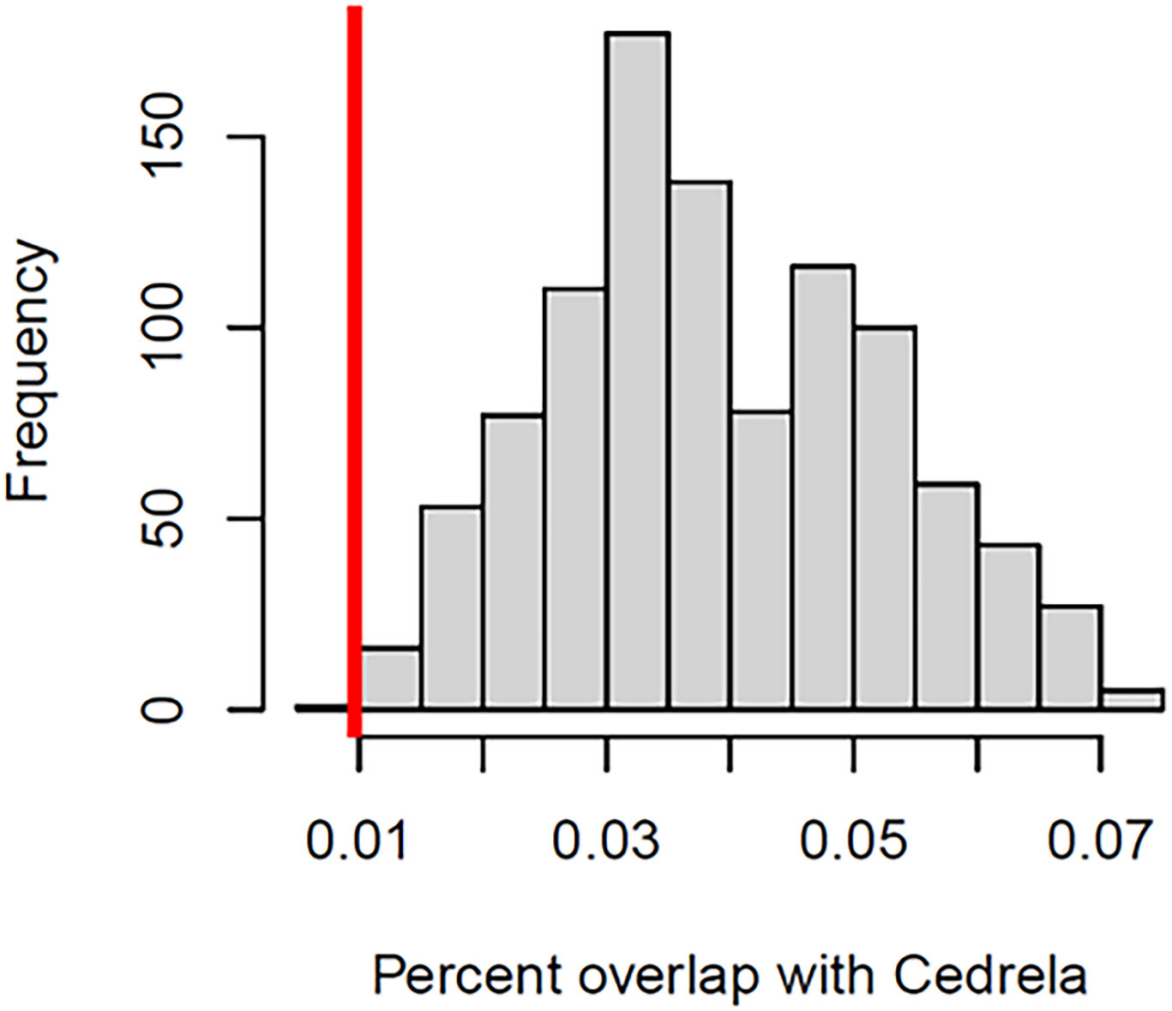
Histogram of percent of overlap of randomized tortoise migration trajectories with *Cedrela* patches in southwestern Santa Cruz Island, Galapagos. These randomized paths provide a null expectation of the potential overlap with *Cedrela* if tortoises were not responding to it. Observed overlap (red line) is significantly lower than these expectations, indicating a clear avoidance of *Cedrela* by tortoises.

The avoidance of *Cedrela* forest by tortoises was remarkable (Figure 2). Of 25 individual tortoises tracked, we found that five tortoises consistently ended their upslope migrations either a short distance (<350 m) into *Cedrela* forest or at the lower edge of a *Cedrela* forest block. (Figure 2 and Table S1). Of the remaining 20 tortoises that traversed the national Park boundary (one individual failed to do so), seven entered the peripheries of *Cedrela* forest. Only three individuals, and 12 (8%) of 140 migratory journeys traversed extensive patches of *Cedrela* forest (Figure 2 and Table S1). The percentage of locations that overlapped with *Cedrela* forest remained low across our randomized paths (Figure 3), which is largely explained because *Cedrela* currently occupies a small fraction of the overall migratory journey of the tortoises (Figure 2). Nevertheless, the observed tortoise trajectories still differed significantly from the null expectation, indicating tortoises significantly avoided *Cedrela* forest during their migrations (*p* = .001, Figure 3).

In short, our analysis demonstrates that WSC tortoise migrations occur almost exclusively through small, restricted corridors where *Cedrela* has yet to invade. The nature of the “*Cedrela* corridors” varies along the national park/farmland border (Figure 2). There are two wide corridors of 1.18 and 0.41 km at their narrowest (labeled C and B in Figure 2) around which both are open and free of *Cedrela*. The third corridor is just 0.14 km at its narrowest (label A in Figure 2) and is characterized by a convoluted set of gaps through the *Cedrela*.

## 4 DISCUSSION

We have shown that WCS tortoises strongly avoid *Cedrela* forest during their migratory journeys. In some cases, where tortoises cannot find a gap in the invasive vegetation, *Cedrela* forest appears to completely block migration. Three migrations terminated at the edge of large *Cedrela* blocks, and two journeys ended a short way into large blocks. Only three of 25 tortoises migrated through the heart of large *Cedrela* forest blocks, and most migratory journeys (12 of 140) were funneled through small gaps between *Cedrela* patches. Further expansion of invasive *Cedrela* is likely to reduce, and ultimately eliminate migration corridors for WCS tortoises. Around the world, barriers that prevent migration are usually devastating for migratory species (Sawyer et al., 2009), and demographic responses of Galapagos tortoises to cessation of migration are likely to be no different. A bioenergetic model indicates that if migrations become blocked, the future energy balance and reproductive output of WSC Galapagos tortoises will be jeopardized (Yackulic et al., 2017). Pervasive negative demographic consequences (i.e., population reduction) will not, however, be obvious for many years due to the longevity and long generation time of this species.

The impact of elimination of WSC tortoise migration will likely permeate beyond species conservation alone and trigger cascading ecological impacts. Galapagos tortoises are ecosystem engineers that play profound roles as herbivores, biological bulldozers, seed dispersers, and nutrient recyclers (Gibbs et al., 2010). These ecological processes shape community ecology over local, and because of the migration, landscape extents (Somveille & Ellis‐Soto, 2022). Blocking the migration will profoundly modify patterns of tortoise engineering with variable ecosystem consequences. For example, limiting the spatial extent of tortoise‐mediated seed dispersal will not only reduce the rate of expansion of numerous invasive plant species, but also prevent the reintroduction of native species dispersed by tortoises where they have gone locally extinct (Blake et al., 2012). If blocking tortoise migration results in a population decline on a similar scale to that observed in many other species to below ecological functionality, even local ecological processes mediated by tortoises will decline and eventually be eliminated.

*Cedrela* invasion is not limited to Santa Cruz Island. Extensive stands now occur along the flanks of the volcanoes of southern Isabela Island where several endemic tortoise species are present. Smaller *Cedrela* forest patches are also present on Santiago and San Cristobal Islands, (Rivas‐Torres, Luke Flory, & Loiselle, 2018), and each island has its own endemic migratory tortoise species. While movement patterns of these endemic tortoise species are not known, based on previous work (Bastille‐Rousseau et al., 2017), elevational migration is likely.

*Cedrela* has biological and ecological traits that make it a highly successful invader and currently one of the most widely distributed invasive trees in Galapagos. *Cedrela* saplings can grow several meters in a few months and flower only 2 years after germination. *Cedrela* has high fecundity, is wind dispersed, is a climate generalist, and is allelopathic, all of which enable it to outcompete many native and even exotic plants (Rivas‐Torres & Rivas, 2018). Predicted increases in rainfall and temperature on Galapagos (Charney et al., 2021) will favor regeneration and range expansion in coming decades (Estrada‐Contreras et al., 2016). All these factors indicate that *Cedrela* will likely continue to expand into native habitats further reducing the opportunities for migration of Galapagos tortoises on Santa Cruz and elsewhere.

Management of *Cedrela* locally and globally is a paradox. *Cedrela* is renowned for its high quality timber which makes it one of the most important species of tropical timber (Mark & Rivers, 2017). *Cedrela* is listed as vulnerable by the IUCN Red List, and, in 2019, because of overharvesting on continental South America, all members of the genus *Cedrela* were placed on Table S1 of CITES. The paradox lies in that *Cedrela* is also listed on the Global Invasive Species Database of the IUCN (Global Invasive Species Database, 2022) because of invasions not just on Galapagos, but also on many Pacific Islands and in South Africa.

The silvicultural and social challenges of managing *Cedrela* on Galapagos are considerable. Given its current range, *Cedrela* eradication and even control in Galapagos might be unfeasible. Furthermore, *Cedrela* wood is the only source of local timber available on the islands used for construction, furniture, and handicrafts for the tourism industry, thus *Cedrela* plays an important role in the local economy. These social and economic values must be factored into future *Cedrela* management planning (Rivas‐Torres & Adams, 2018).

Finally, the Galapagos National Park is under heavy local, national, and international scrutiny; thus, decisions on the future management of *Cedrela* must involve options based on strong science, with high stakeholder support from local to global scales (Rivas‐Torres & Adams, 2018). The complexity of these issues means that a brief set of recommendations cannot do justice to the scale of the problems; however, given the urgency facing the future of tortoise migrations on Sant Cruz Island, some immediate priorities are worth consideration:

1. Given the locally destructive nature of *Cedrela*, the legal framework must be revised to allow for the establishment of a proactive management strategy for the Galapagos Archipelago. The local legal framework should not compromise international conservation efforts and existing international legal structures for this species.
2. An integrated research agenda should be established to: (a) model the rate of expansion of *Cedrela* over time since its introduction to Galapagos and predict its current rate of expansion into tortoise migration corridors as well as assess limits to its distribution under climate change scenarios, (b) understand the impact of different harvest regimes and silviculture methods on future vegetation dynamics and a cost/benefit/risk assessment of eradication and other management scenarios (e.g., containment and mitigation), and (c) develop plans for utilization of *Cedrela* under management and/or eradication scenarios and the future of the timber industry of Galapagos.
3. Convene an expert/stakeholder workshop to consider priority management actions required to maintain tortoise migrations in the short to medium term and develop the mechanisms to achieve recommendations 1 and 2 above.

## AUTHOR CONTRIBUTIONS

**Stephen Blake:** Conceptualization (lead); data curation (lead); formal analysis (equal); funding acquisition (lead); investigation (lead); methodology (lead); project administration (lead); resources (lead); writing – original draft (lead); writing – review and editing (lead). **Freddy Cabrera:** Conceptualization (supporting); data curation (supporting); investigation (equal); methodology (equal); project administration (equal). **Gonzalo Rivas‐Torres:** Data curation (supporting); methodology (supporting); writing – review and editing (supporting). **Sharon L. Deem:** Funding acquisition (supporting); investigation (supporting); project administration (equal); writing – review and editing (supporting). **Ainoa Nieto‐Claudin:** Data curation (supporting); project administration (supporting); supervision (supporting); writing – review and editing (supporting). **Rakan A. Zahawi:** Project administration (supporting); supervision (supporting); writing – review and editing (supporting). **Guillaume Bastille‐Rousseau:** Conceptualization (supporting); formal analysis (equal); methodology (supporting); writing – review and editing (supporting).

## FUNDING INFORMATION

This project was supported by the National Science Foundation (DEB 1258062), The Max Planck Institute for Animal Behavior (Radolfzell, Germany), the National Geographic Society Committee for Research and Exploration, the Galapagos Conservation Trust, Houston Zoo, the Saint Louis Zoo, Institute for Conservation Medicine and WildCare Institute, the Woodspring Trust, and the Swiss Friends of Galapagos.

## CONFLICT OF INTEREST STATEMENT

The authors declare no conflict of interest.

## Supporting information


Table S1.

## Data Availability

The *Cedrela* shapefile used in this article from the Vegetation Map of the Galapagos v.2016, is available from The Plant Ecology Lab https://ecologyecuador.com. Galapagos tortoise movement data are available through www.movebank.org within the Galapagos Tortoise Movement Ecology Programme study. Both datasets are available for peer review on Dryad https://datadryad.org/stash/share/aHFtc‐aYQe0pR9xKFrEtQQZlc5aOeCpdVtdj0la2qBg, and will be made publicly available upon acceptance of this article for publication.
