# Supplementary material for: Invasion by Cedrela odorata threatens long distance migration of Galapagos tortoises
Source: Ecol Evol. 2024 Feb 13;14(2):e10994. doi: 10.1002/ece3.10994 (PMC10864728; doi:10.1002/ece3.10994)
Supplement: Supplementary file 1 — Table S1. [file ECE3-14-e10994-s001.docx]

Table S1

**Table S1. Summary statistics of duration of GPS telemetry for a sample of migratory Western Santa Cruz Galapagos tortoises, including details of association with *Cedrela* Forest**

| Tortoise sex | Date of first fix | Date of last fix | N days tracked | N GPS fixes | Terminated migration at/in *Cedrela* forest patch | Crossed National Park boundary | Crossed *Cedrela* Forest periphery | Migrated through *Cedrela* forest block |
| --- | --- | --- | --- | --- | --- | --- | --- | --- |
| Female | 1-Aug-13 | 27-Nov-15 | 849 | 1519 |  | Yes |  |  |
| Female | 1-Feb-19 | 23-Oct-22 | 1360 | 66202 |  | Yes |  |  |
| Female | 11-Jul-13 | 12-May-17 | 1401 | 25350 | Yes |  |  |  |
| Female | 29-Jan-19 | 18-Jan-21 | 720 | 67632 |  | Yes |  |  |
| Female | 14-Feb-20 | 29-Jan-21 | 351 | 26087 |  | Yes |  |  |
| Female | 14-Sep-10 | 16-Mar-17 | 2375 | 31494 |  | Yes | Yes | Yes |
| Female | 9-Jul-13 | 30-Jul-17 | 1482 | 31763 |  | Yes | Yes | Yes |
| Female | 14-Sep-10 | 19-Sep-16 | 2196 | 18328 |  | Yes | Yes |  |
| Female | 10-Dec-09 | 19-Jan-12 | 771 | 12188 |  | Yes |  | Yes |
| Female | 12-Jul-13 | 7-May-16 | 1031 | 1876 | Yes |  |  |  |
| Female | 1-Aug-13 | 4-May-14 | 276 | 483 | Yes |  |  |  |
| Female | 26-Jul-13 | 27-Oct-14 | 459 | 852 | Yes |  |  |  |
| Female | 18-Feb-14 | 11-Dec-15 | 662 | 10271 | Yes |  |  |  |
| Female | 9-Jul-13 | 9-Feb-16 | 945 | 20032 |  | Yes | Yes |  |
| Female | 9-Jul-13 | 17-Jul-16 | 1104 | 18333 |  | Yes | Yes |  |
| Male | 14-Aug-13 | 22-Nov-13 | 101 | 174 |  | Yes |  |  |
| Male | 15-Sep-10 | 2-Aug-13 | 1052 | 14359 |  | Yes |  |  |
| Male | 23-Dec-19 | 11-Jan-22 | 750 | 17871 |  | Yes |  |  |
| Male | 8-Aug-19 | 25-Aug-22 | 1114 | 24610 |  | Yes |  |  |
| Male | 14-May-09 | 29-Dec-14 | 2054 | 51320 |  | Yes | Yes |  |
| Male | 14-Sep-10 | 29-Jan-15 | 1598 | 23711 |  | Yes |  |  |
| Male | 4-Jun-12 | 30-Nov-15 | 1274 | 18557 |  | Yes |  |  |
| Male | 7-Jul-20 | 27-Jul-22 | 750 | 13543 |  | Yes |  |  |
| Male | 20-Sep-10 | 23-Jul-21 | 3959 | 54025 |  | Yes | Yes |  |
| Male | 12-Dec-09 | 12-Mar-12 | 822 | 13392 |  | Yes |  |  |
| Total |  |  | 29601 | 564227 |  |  |  |  |
